# Supplementary figures and images for: The effective family size of immigrant founders predicts their long-term demographic outcome: From Québec settlers to their 20th-century descendants
Source: PLoS One. 2022 May 4;17(5):e0266079. doi: 10.1371/journal.pone.0266079 (PMC9067642; doi:10.1371/journal.pone.0266079)

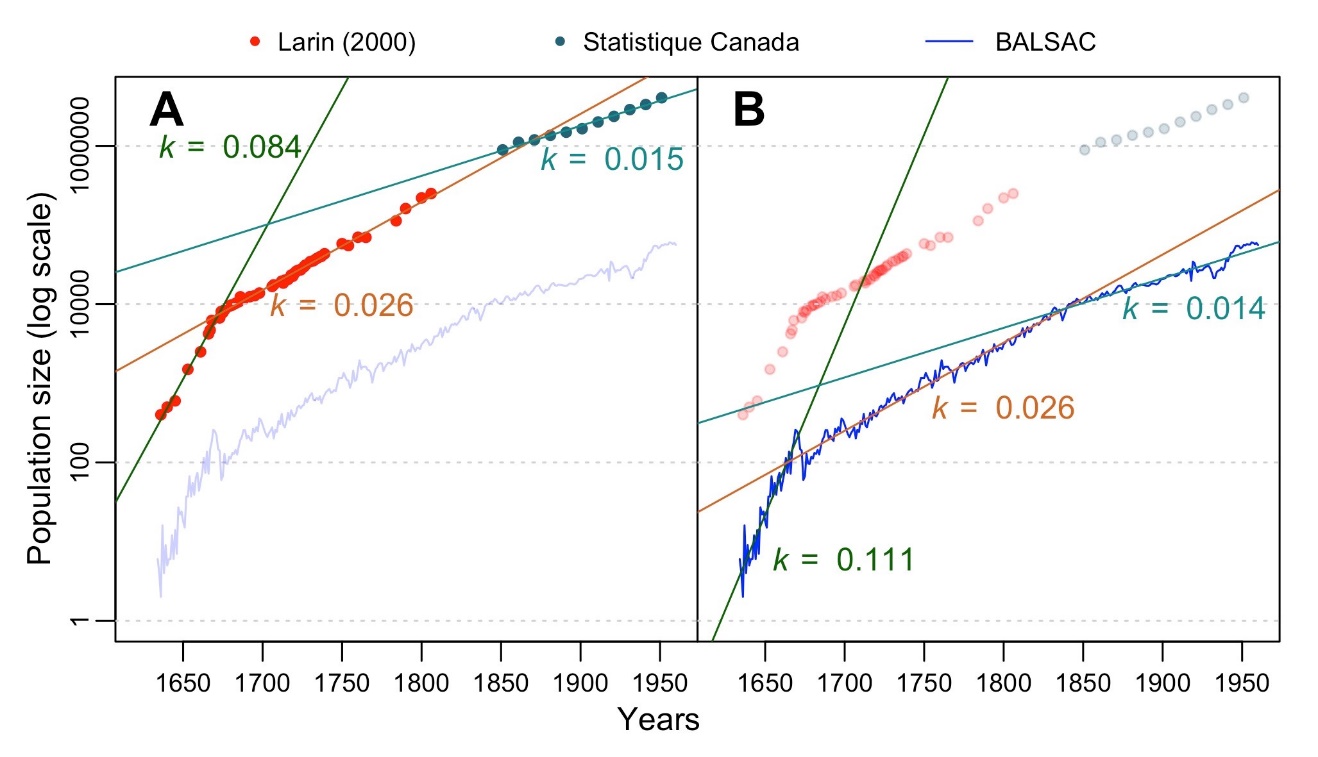

Supplement: S2 Fig — Growth rate (k) is estimated from the slope of the plot of historical population size versus time. In (A), we used population size data reported by Larin (red circles) ([31] and references therein) and Statistics Canada census data since 1851 (green circles) (http://www.stat.gouv.qc.ca/default_an.html). In (B), to estimate k, we used the yearly number of BALSAC recorded marriages (blue line–shown in pale blue in (A)) as a proxy for the population size. Linear regression curves were calculated by fitting the log of population sizes to the years by periods: 1621–1670 in dark green, 1671–1850 in brown and 1851–1960 in cyan. The associated slope coefficients (k) are displayed using the same color code. (DOCX) [file pone.0266079.s002.docx]

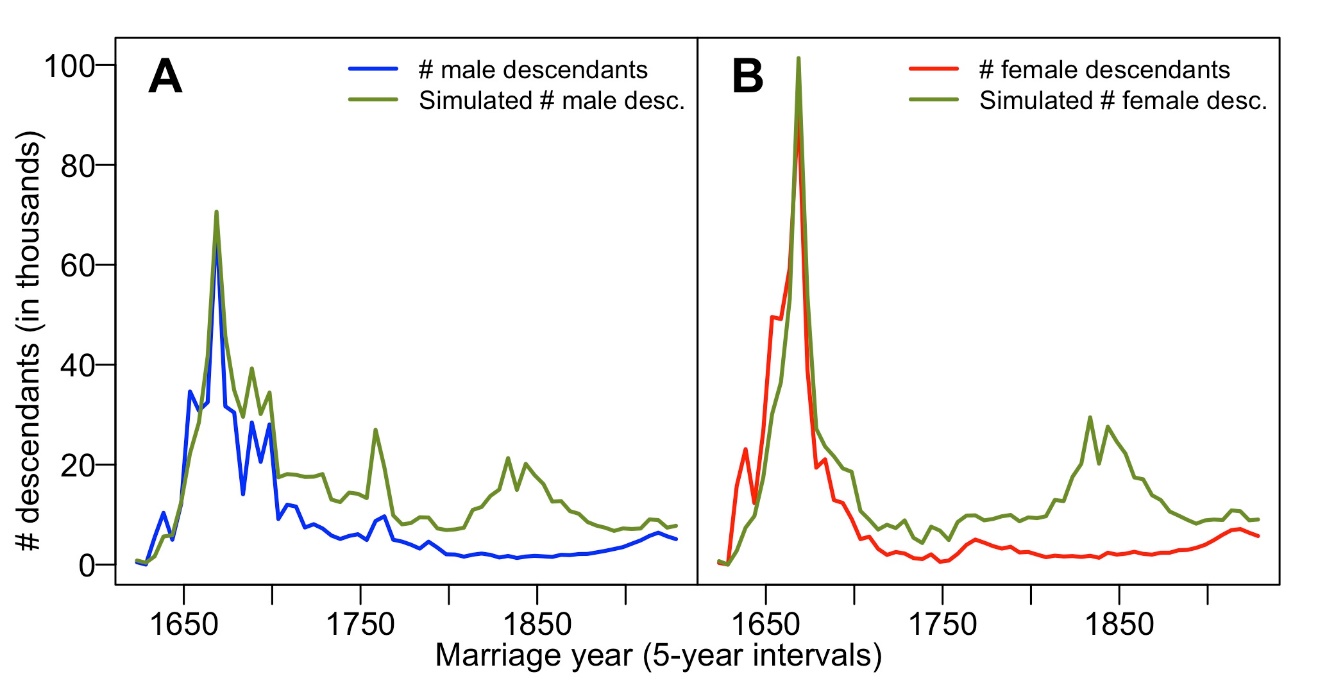

Supplement: S3 Fig — In (A), paternal lineages with the observed (blue) and simulated (green) numbers of male descendants. (B) Maternal lines with the observed (red) and simulated (green) numbers of female descendants. (DOCX) [file pone.0266079.s003.docx]

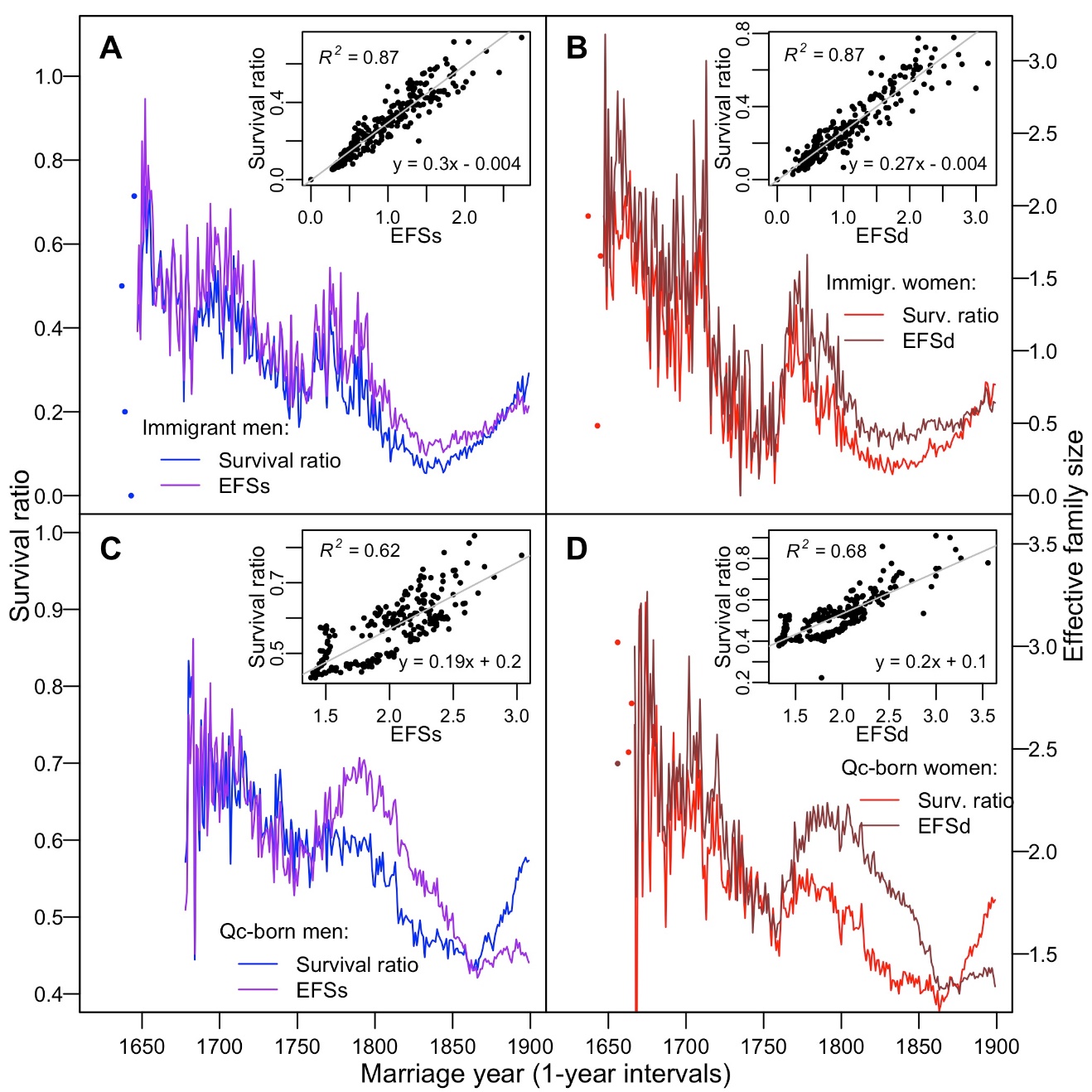

Supplement: S4 Fig — Overlapping plots of total EFSs and EFSd and their survival ratios for the immigrants (A and B) and the Quebec-born lineages (C and D). Their correlation plots are shown in the insets. (DOCX) [file pone.0266079.s004.docx]

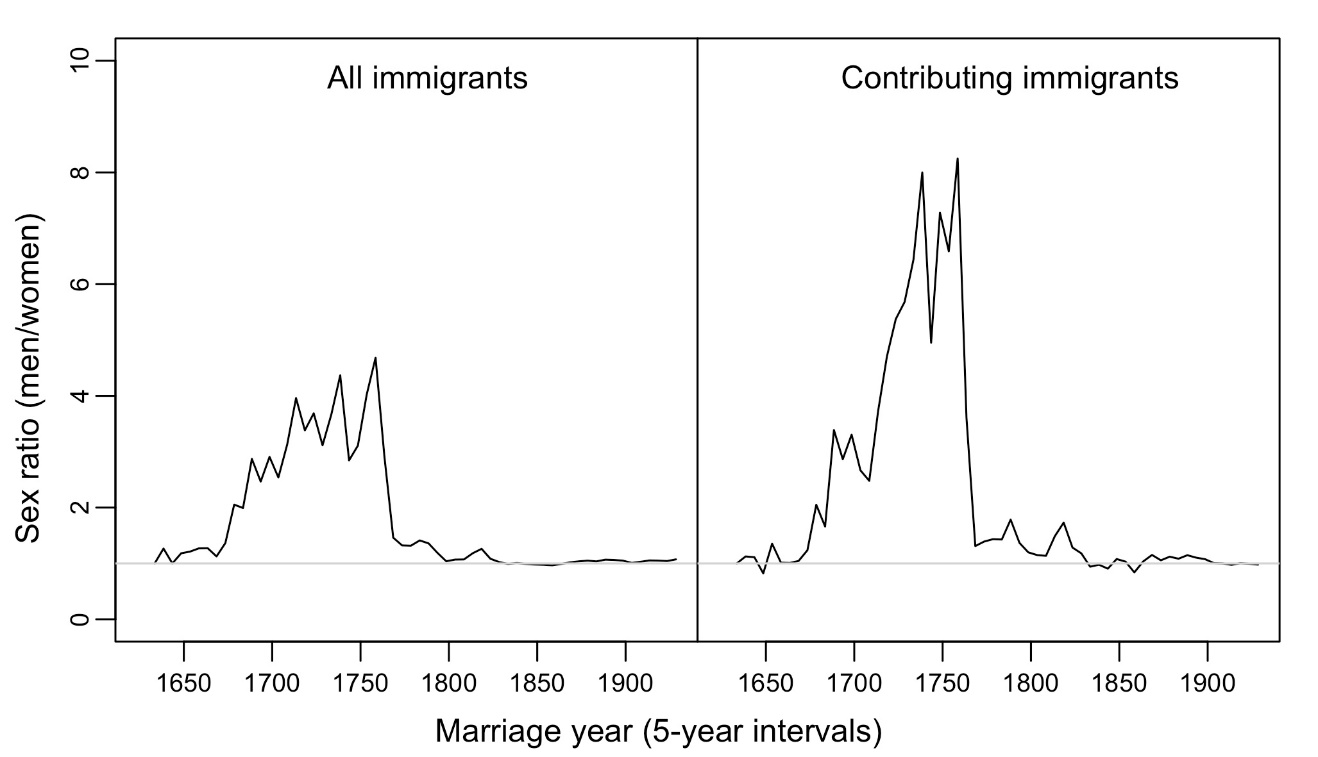

Supplement: S5 Fig — The number of male immigrants divided by the number of female immigrants considering all immigrants (left panel) and only a subset of the contributing ones (right panel). (DOCX) [file pone.0266079.s005.docx]

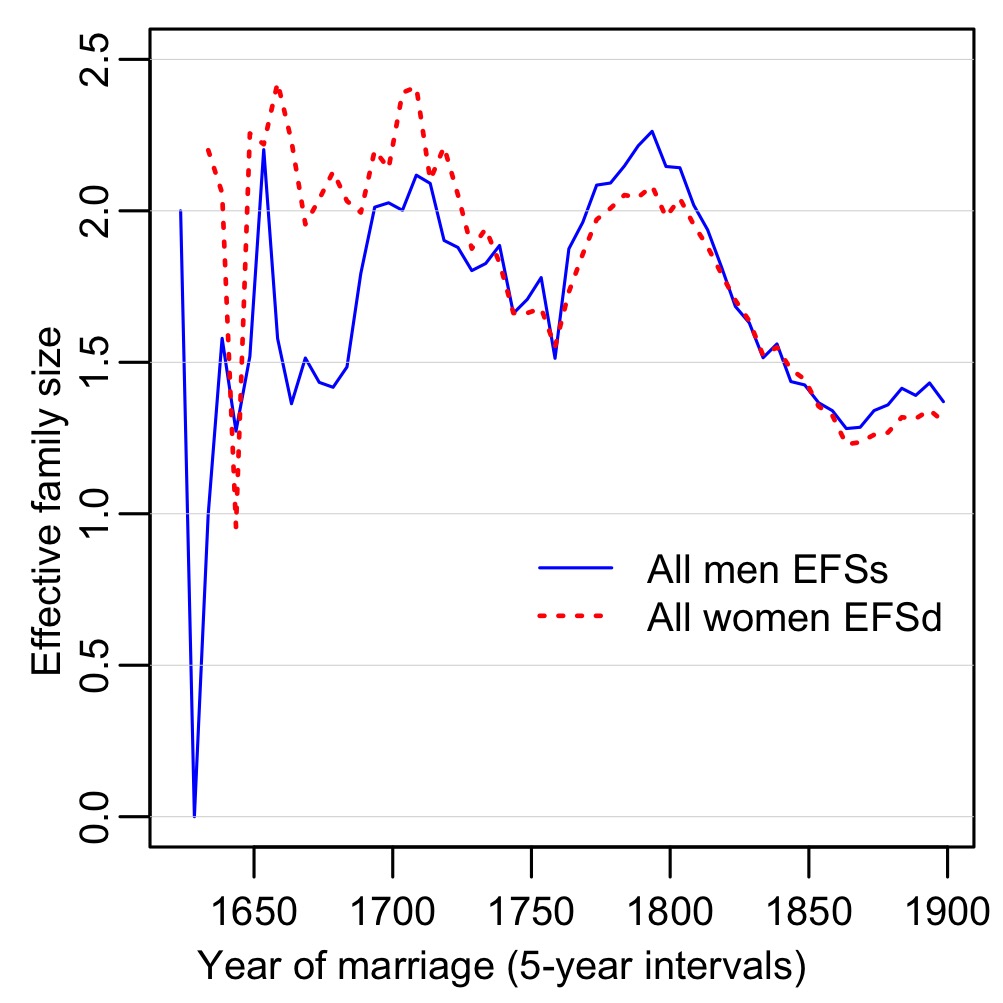

Supplement: S6 Fig — Please, note much lower EFS-sons (EFSs) than that of EFS-daughters (EFSd) before 1700, at the second half of the 17th century. This is consistent with the scenario of many Québec-born men, from immigrant and non-immigrant parents at that time, to leave Québec exploring other territories referred to as Nouvelle France. (DOCX) [file pone.0266079.s006.docx]

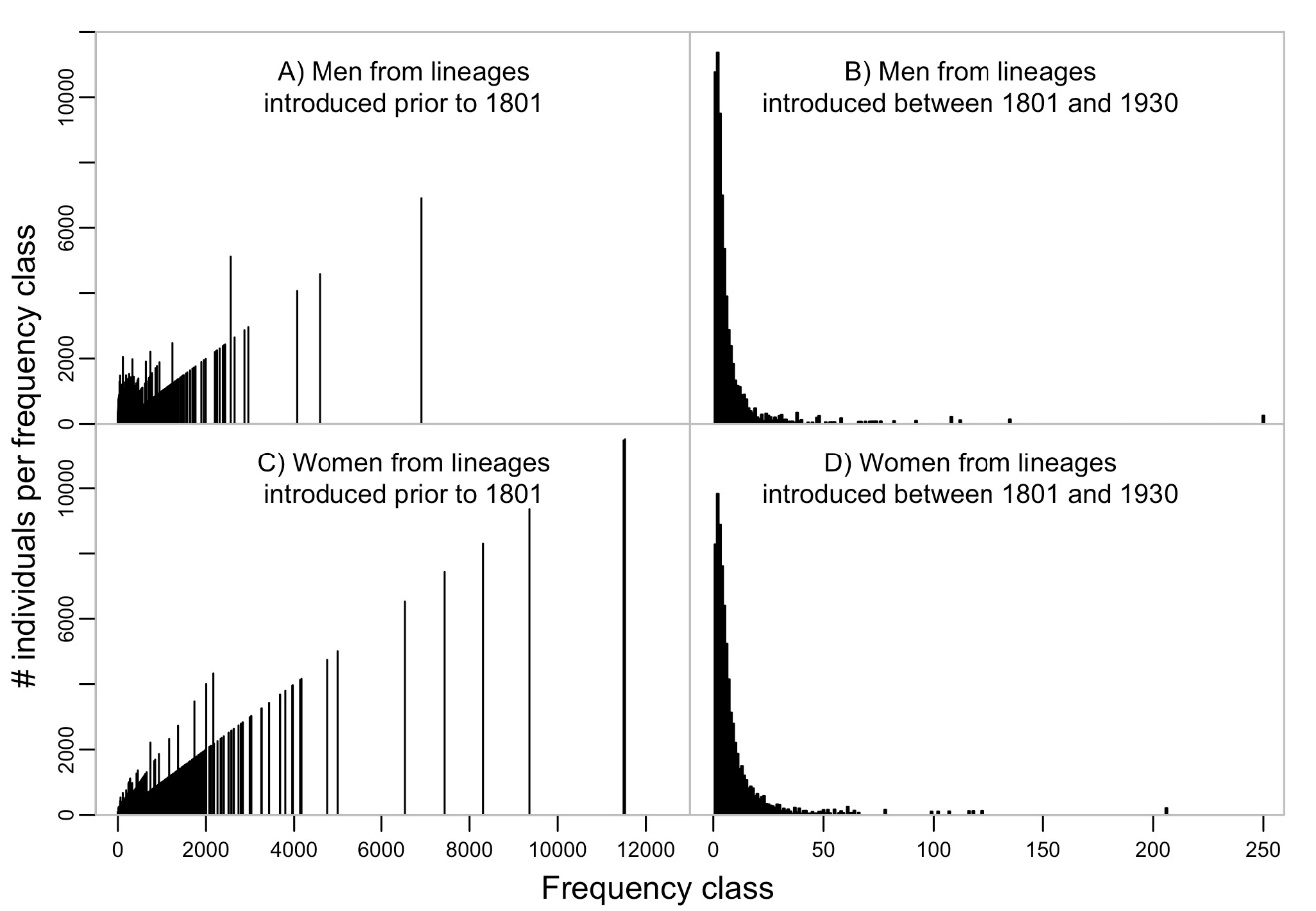

Supplement: S7 Fig — The lineages introduced before 1801 are shown in the left panels, and those introduced between 1801 and 1930 are in the right plots. Note that on the y-axis, we present the whole number of individuals within the frequency class to make the plot more informative and transparent as in (41). A typical plot of frequency classes registers only the number of classes on the y-axis. As a result, frequency classes represented only a few times or once, practically disappear from graphic representation, especially when frequency classes on the left are particularly numerous. (DOCX) [file pone.0266079.s007.docx]

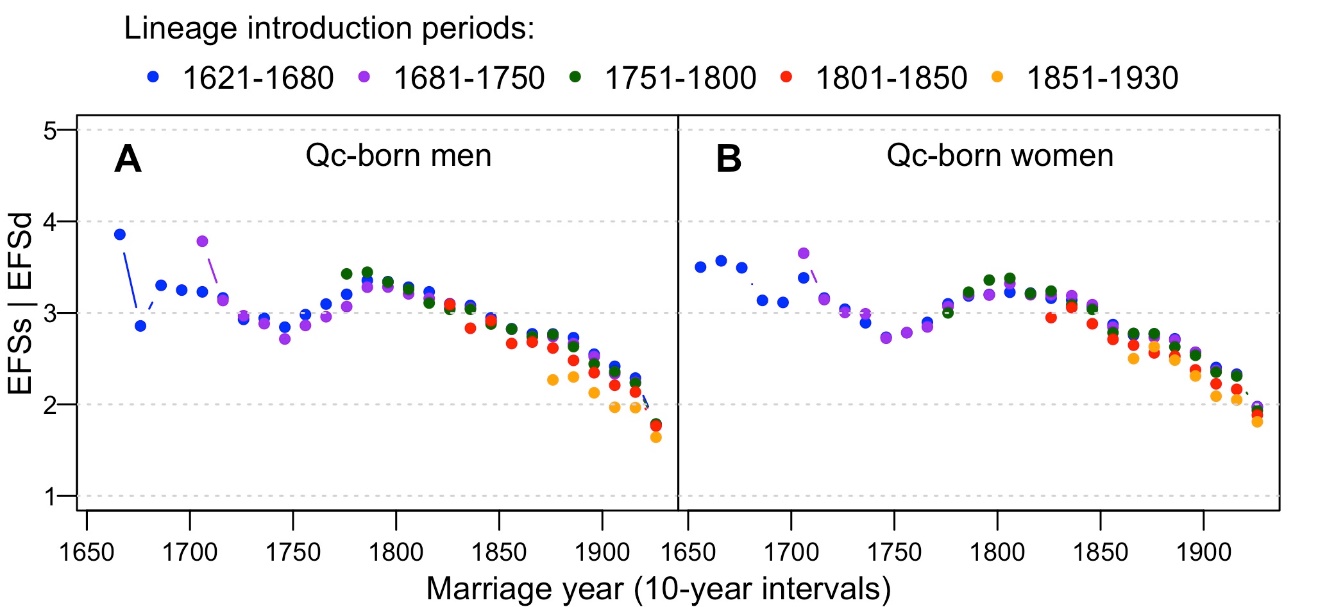

Supplement: S8 Fig — EFS is averaged over 10-year intervals, and progeny of lineages at different periods are marked by different colors as indicated. Remember that contributing refers to lineages still present within the 1931–60 Québec population. (DOCX) [file pone.0266079.s008.docx]

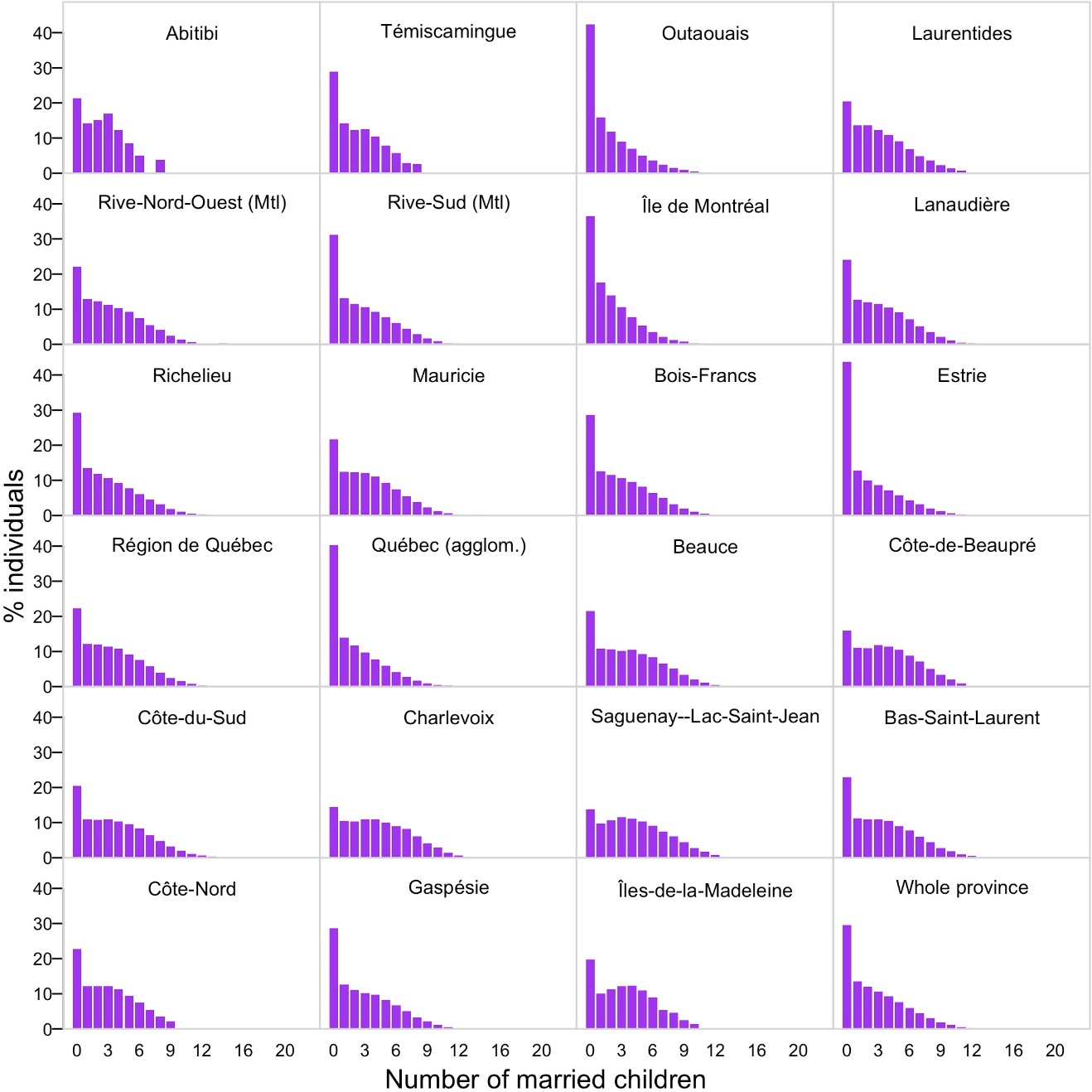

Supplement: S9 Fig — Because only children married in Québec (registered in BALSAC) are counted, it emphasizes the potential effect of emigration out of Québec on the regional EFS frequency distribution. Note that "zero frequency EFS" exceeds the 10–15% threshold well, ascribed to couple sterility in many regions. Likewise, convex histograms of subsequent frequency classes, such as in Charlevoix, become concave when preceded by high zero EFS frequency, as in Outaouais or Isle de Montréal. Emigration affects the EFS of all families. Besides emigration (strictly, leaving Québec and married or not elsewhere), other factors: Economic, social, and historical (e.g., wars, epidemics) could have reduced BALSAC recorded EFS. (DOCX) [file pone.0266079.s009.docx]

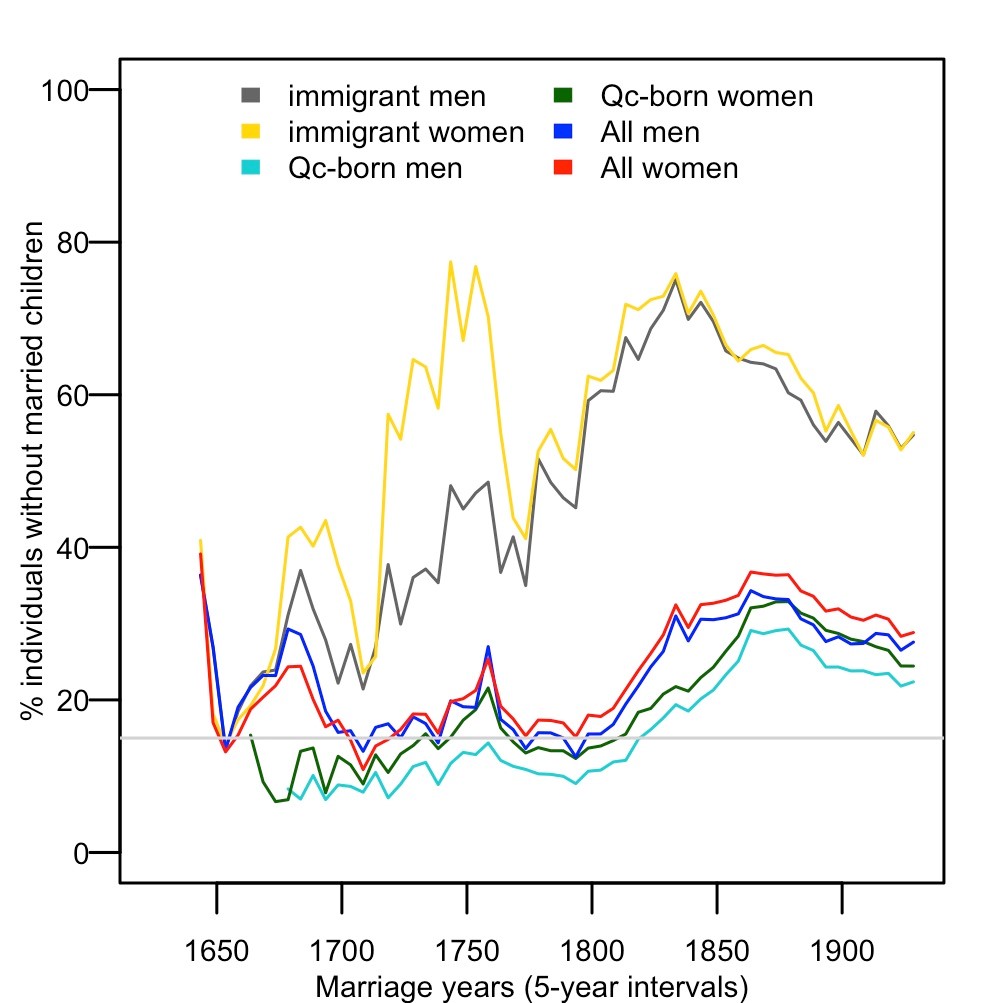

Supplement: S10 Fig — Percentages of immigrant men (dark grey line), immigrant women (yellow line), QB men (cyan line), QB women (green line), all men (blue line), and all women (red line) without married children as a function of their marriage year by 5-year intervals. The light grey line indicates an estimate of the infertility rate (15%; [58]). (DOCX) [file pone.0266079.s010.docx]
