# Supplementary material for: The effective family size of immigrant founders predicts their long-term demographic outcome: From Québec settlers to their 20th-century descendants
Source: PLoS One. 2022 May 4;17(5):e0266079. doi: 10.1371/journal.pone.0266079 (PMC9067642; doi:10.1371/journal.pone.0266079)
Supplement: Supplementary file 2 [file pone.0266079.s001.docx]

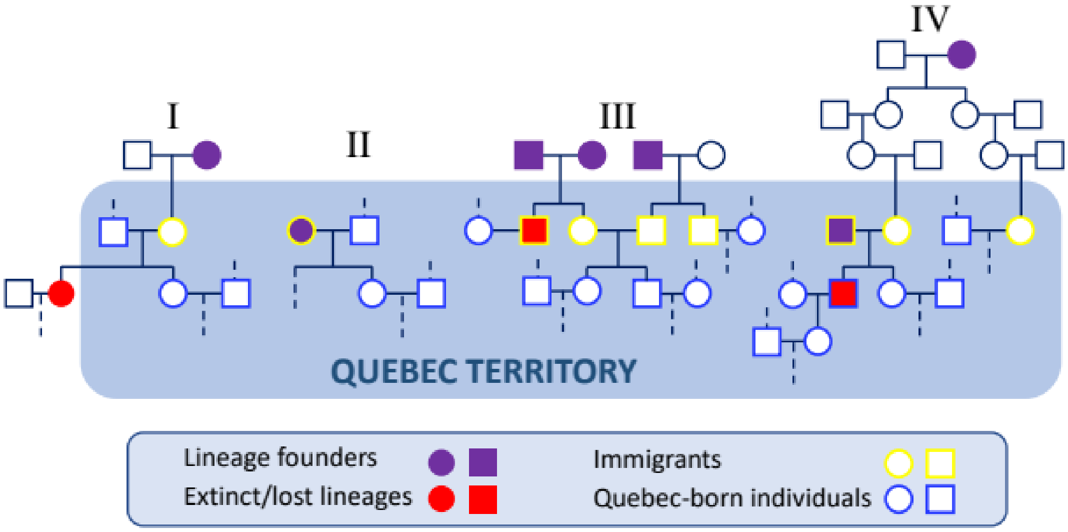
I
II
III
IV
QUEBEC TERRITORY
Lineage founders
Extinct/lost lineages
Immigrants
Quebec-born individuals
