# Supplementary material for: The effective family size of immigrant founders predicts their long-term demographic outcome: From Québec settlers to their 20th-century descendants
Source: PLoS One. 2022 May 4;17(5):e0266079. doi: 10.1371/journal.pone.0266079 (PMC9067642; doi:10.1371/journal.pone.0266079)
Supplement: Attachment — Submitted filename: Plos One Review Comments.docx [file pone.0266079.s011.docx]

Plos One Review

Line 23 - Not sure what it is meant with “we followed their transmission in real-time” within this context. Please explain or rephrase.

Line 55 – Iceland, not Island.

Line 61 – What constitutes “essential characteristics”? On one hand it seems that we cannot be without this type of information and on the other hand there is not mention of what it is.

Line 67 – Genealogical lineages

Line 68 – mitochondrial DNA (mtDNA) 🡪 since this is the first instance of mentioning it and afterwards (see line 73) it is abbreviated.

Line 68 and 73 – Uniparentally-inherited (see line 180)

Line 68 – the non-recombining

Line 75 – Note: although it is correct to state that mtDNA and Ycs are genetically different (“genetic diversities”), I believe what the authors really meant is that they also reflect a different genetic history: routes, dispersion, roles, etc. in retelling the history of humankind. This in addition to the different mutation rates they each have.

Line 102 – The formatting for the parenthesis need to be fixed

Table 2 – Fix number of Married Man 1751-1800 (5 0064)

Line 329 – coalescent theory?

Line 350 – “their descending marrieds recorded” – what is it?
